# Deafness for High Notes

**Published:** 1891-03

**Authors:** 


### Deafness for High Notes.

Mr. Edwin Cowles, editor of the *Cleveland Leader,* who died last March, had a peculiar form of deafness. He never heard the sound of a bird's note, and until he grew to manhood he always thought the music of the bird was a poetical fiction. "You may fill the room with canary birds," he once said, "and they may all sing at once, and I would never hear a note, but I would hear the fluttering of their wings. I never heard the hissing sound in the human voice; consequently, not knowing of the existence of that sound, I grew up to manhood without ever making it in my speech. A portion of the consonants I never hear, yet I can hear all the vowels. About a quarter of the sounds in the human voice I never hear, and I have to watch the motion of the lips and be governed by the sense of the remarks in order to understand what is said to me. I have walked by the side of a policeman going home at night and seen him blow his whistle and I never could hear it, although it could be heard by others half a mile away. I never heard the upper notes of the piano, violin, or other musical instruments, although I would hear all the lower notes."—*Cleveland Med. Gaz.*

